# Different Peripheral Tissue Injury Induces Differential Phenotypic Changes of Spinal Activated Microglia

**DOI:** 10.1155/2013/901420

**Published:** 2013-05-29

**Authors:** Kai Li, Yong-Hui Tan, Alan R. Light, Kai-Yuan Fu

**Affiliations:** ^1^Center for TMD & Orofacial Pain, Peking University School & Hospital of Stomatology, Zhong Guan Cun South Avenue 22, Beijing 100081, China; ^2^Department of General Dentistry II, Peking University School & Hospital of Stomatology, Beijing 100081, China; ^3^Department of Anesthesiology, University of Utah, Salt Lake City, UT 84132-2304, USA

## Abstract

The purpose of this study is to investigate the possible different cellular marker expression associated with spinal cord microglial activation in different pain models. Immunohistochemistry and western blotting analysis of CD45, CD68, and MHC class I antigen as well as CD11b and Iba-1 in the spinal cord were quantitatively compared among widely used three pain animal models, complete Freund's adjuvant (CFA) injection, formalin injection, and chronic constriction injury (CCI) models. The results showed that significant upregulated expressions of CD45 and MHC class I antigen in spinal microglia as well as morphological changes with increased staining with CD11b and Iba-1 were seen in CCI and formalin models and not found in CFA-induced inflammatory pain model. CD68 expression was only detected in CCI model. Our findings suggested that different peripheral tissue injuries produced differential phenotypic changes associated with spinal microglial activation; peripheral nerve injury might induce spinal microglia to acquire these immunomolecular phenotypic changes.

## 1. Introduction

Studies in recent decades implicate that not only neurons but also glial cells (microglia and astrocytes) play important roles in the generation and maintenance of pathologic pain [1–6]. Both microglia and astrocytes are activated in the spinal cord in almost all animal models of pain, including nerve injury, traumatic injury, inflammatory, and bone cancer pain models [7–16]. However, the molecular mechanisms by which glial cells are activated and contribute to the pain situation are not fully understood. Microglial cells are the resident macrophages and the local immune cells in the central nervous system (CNS). Immune responses particularly through the activity of microglia in the spinal cord contribute to pain behaviors after injury to the nervous system [17]. Intrathecal treatment with low-dose methotrexate (an immunosuppressive drug) reduces microglial activation and attenuates pain-like behavior after nerve injury [18, 19]. 

Microglia are quickly activated following peripheral nerve injury or tissue inflammation [20, 21] and begin to upregulate cell surface molecules and express cytokines, chemokines, and effector molecules. Microglia are highly dynamic and undergo different cellular remodeling throughout the progression of diseases and in distinct pathological conditions [22]. In the normal CNS, microglia are characterized by ramified processes arising from a relatively small cell body, with a weak expression of molecules normally expressed by other hematopoietic lineages. It is well known that certain immune molecules, including CD11b/c, CD45, CD40, CD86, major histocompatibility complex (MHC) class II, which are markers of both macrophage and dendritic lineage cells [23], as well as Toll-like receptor, Fc receptor, and cytokine receptors [24–27], are significantly upregulated on activated microglia in a variety of experimental and clinical CNS diseases. Little is known about the expression of these immune molecules in activated microglia in different pain models [28–30]. We previously demonstrated upregulated expression of CD45 and MHC class I antigens in activated microglia along with significant morphological changes starting on day 1–3 and peaking on day 7 after peripheral formalin injection [28]. The expression of MHC class II, CD11c, and the Fc receptor was maintained at a low basal level and did not change after the stimuli.

To understand the way activated microglia contribute to the pathogenesis of different induced pain, we compared three widely used pain models comprising injection of complete Freund's adjuvant (CFA) into the hind paw which is a predominantly inflammatory pain model, subcutaneous injection of dilute formalin into the hind paw which is a mixed inflammatory and nerve injury model, and the chronic constriction nerve injury (CCI) model which is a neuropathic pain model. The difference on microglial morphological changes between formalin and CFA model has already been detected in our previous study [31]. The aim of the present study is to address the expression level of immune molecules CD45, CD68, and MHC class I, as well as microglial markers CD11b and Iba-1, using both immunofluorescence and western blot.

## 2. Materials and Methods

### 2.1. Animals Models

Adult male Sprague-Dawley rats weighing 200–225 g (Vital River Laboratory Animal Technology Co., Ltd., Beijing) were used. All rats were housed at a temperature of 23°C (±1°C) on a 12-hour light/dark cycle with free access to food and water. The experimental protocols were approved by the Animal Care and Use Committee of Peking University Health Science Center. The care and use of animals conformed to applicable national/international guidelines. 

Fifty-six rats were randomly assigned to four groups (8 rats per group). CCI rats were produced by the loose ligation of the common sciatic nerve on the right side according to the method of Bennett and Xie [32]. Briefly, the right sciatic nerve was exposed at the mid-thigh level and separated from the adjacent tissue. Four loose ligatures using 4–0 chromic gut sutures were made around the dissected nerve with about 1.0 mm interval between each pair of ligatures. The skin wound was closed with sterile silk sutures. The surgical procedure was performed under pentobarbital anesthesia (40 mg/kg, i.p., supplemented as necessary). The formalin injected rats received subcutaneous injections of 100 *μ*L 5% formalin (diluted in 0.9% saline) into the plantar surface of the right hind paw. CFA injected rats were injected subcutaneously into the right hind paw with 100 *μ*L CFA (Sigma-Aldrich) mixed 1 : 1 with normal saline. Age-matched naïve rats with no treatment were used as the control group. 

### 2.2. Immunohistochemistry

Animals (4 rats for each group) were anesthetized on day 7 following the treatments as above with an overdose of pentobarbital sodium, and the rats were euthanized by transcardiac perfusion (250 mL body temperature 0.1 M PBS pH 7.4 followed by 300 mL ice-cold 4% paraformaldehyde/4% sucrose in 0.1 M PB pH 7.4). After perfusion, the lumbar spinal cords (L4–5) were removed, postfixed in 4% paraformaldehyde fixative for 4–6 hours, and placed in a 30% sucrose solution (in 0.1 M PB) over two nights at 4°C. Thirty-micron-thick tissue sections were cut transversely on a cryostat for free-floating immunohistochemical staining for OX-42 (monoclonal mouse anti-rat CD11b, 1 : 200, Serotec, Oxford, UK), Iba-1 (rabbit polyclonal anti-Iba-1, 1 : 400, Abcam, Cambridge, MA), MHC class I antigen (monoclonal mouse anti-rat MHC class I, 1 : 400, Serotec), CD45 (monoclonal mouse anti-rat CD45, 1 : 200, Serotec), and CD68 (monoclonal mouse anti-rat CD68, 1 : 200, Serotec). All of the sections were blocked with 5% normal goat serum in 0.3% Triton X-100 for 1 h at room temperature (RT) and incubated for 48 h at 4°C with the primary antibody. Next, the sections were incubated for 90 min at RT with a corresponding FITC-conjugated secondary antibody. For double labeling, tissues from CCI rats were incubated with a mixture of primary antibody CD45 or CD68 or MHC class I with rabbit anti-Iba-1 (microglia marker, 1 : 2000, Wako), rabbit anti-GFAP (astrocyte marker, 1 : 1000, Chemicon), and rabbit antineutron-specific enolase (NSE, neuronal marker, 1 : 1000, Chemicon). The single-, or double-, stained images were captured with a CCD spot camera and processed using Adobe Photoshop. To analyze MHC class I immunoreactivity, the immunofluorescence intensity was measured as described in our previous reports [9, 31]. The medial portion of the spinal cord dorsal horn was outlined as an area of interest. The average percentage of area covered by MHC class I-immunoreactive (IR) profiles relative to the total outlined area of interest was calculated from three selected tissue sections for each animal. The immunoreactivity level was expressed as the fold increase compared to controls. The rats' sections from all groups were always processed together for comparison under the same conditions.

### 2.3. Western Blot

Rats (4 rats for each group) were deeply anesthetized and decapitated. The spinal cord segments (L4-5) ipsilateral to the treatment were removed rapidly and homogenized in a RIPA lysis buffer (cell signaling technology; supplemented with 1 mM PMSF, phosphatase, and protease inhibitor cocktail, Sigma). The homogenate was centrifuged at 15,000 g for 40 min at 4°C. The protein concentration of tissue lysates was determined with a BCA Protein Assay Kit (Pierce, Rockford, IL). Fifty microgram aliquots were subjected to SDS-PAGE, and the proteins were electrophoretically transferred to PVDF filters (Millipore, Bedford, MA). After being blocked with 5% nonfat milk in Tris-buffered saline (TBS) containing 0.1% Tween-20 for 1 h at room temperature, the membranes were incubated with an anti-Iba-1 (1 : 1000, in 5% BSA, Wako, Osaka, Japan), anti-CD45 (1 : 200, in 5% BSA, BD Bioscience, San Jose, CA), CD68 (1 : 200, Serotec), and *α*-tubulin (1 : 1000, Abcam) antibody overnight at 4°C. After washing, the antibody-protein complexes were probed with HRP-conjugated secondary antibody (1 : 10000, Jackson ImmunoResearch, West Grove, PA), developed in ECL solution for 3 min, and exposed onto Kodak hyperfilms. The density of the immunoreactive bands was quantified using NIH ImageJ 1.38 software (NIH, Bethesda, MD), normalized to the density of internal control (e.g., Iba-1/*α*-tubulin), and expressed as the fold change relative to control group.

### 2.4. Statistical Analysis

MHC class I immunoreactivity and Iba-1, CD45, and CD68 protein levels were statistically analyzed. All data were reported as the mean ± SEM. Differences between groups were compared by ANOVA followed by Tukey post hoc analysis (multiple groups). The criterion for statistical significance was *P* < 0.05.

## 3. Results

### 3.1. Morphological Changes of Microglial Activation, as well as Increased Expression of the Iba-1 Protein Level, Occur on Day 7 after CCI Injury and Formalin Injection, not Evident in CFA Animals

**Figure 1:**
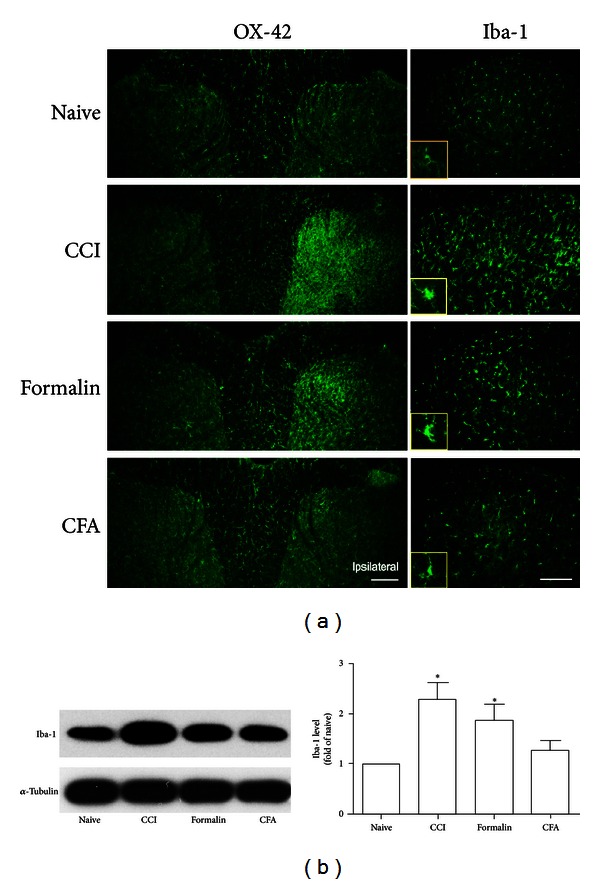
OX-42 and Iba-1 immunoreactivity in the spinal cord dorsal horn after different treatment to rats. A significant increase of OX-42 and Iba-1 expression was present following CCI injury and formalin injection, which was accompanied with obvious morphological change compared with naïve group ((a); Scale bar, 200 *μ*m). The magnified images in yellow box showed the morphology of a single-activated microglia. Representative bands and quantification of western blot analysis (b) showed increased Iba-1 protein level in the lumbar spinal cord after CCI injury and formalin injection, not after CFA injection. *P < 0.05, one-way ANOVA and Tukey post hoc test, compared with the naïve (no treatment) control, *n* = 4.

In naïve rats, microglia labeled by OX-42 and Iba-1 were homogeneously distributed throughout the spinal gray and white matter with a ramified morphology, appearing to be in a resting state (Figure 1(a)). CCI nerve injury and peripheral formalin injection, but not CFA treatment, induced a significant increase of OX-42 and Iba-1 immunoreactivity in the ipsilateral side with morphological changes being a state of clearly hypertrophic (Figure 1(a)). The immunofluorescence result was confirmed by increased Iba-1 protein level demonstrated by Western blot (Figure 1(b)). 

### 3.2. CCI Nerve Injury, Not CFA Injection, Induced Spinal Microglia Upregulation of Immune Molecules, Including CD45, CD68 and MHC I Antigens

**Figure 2:**
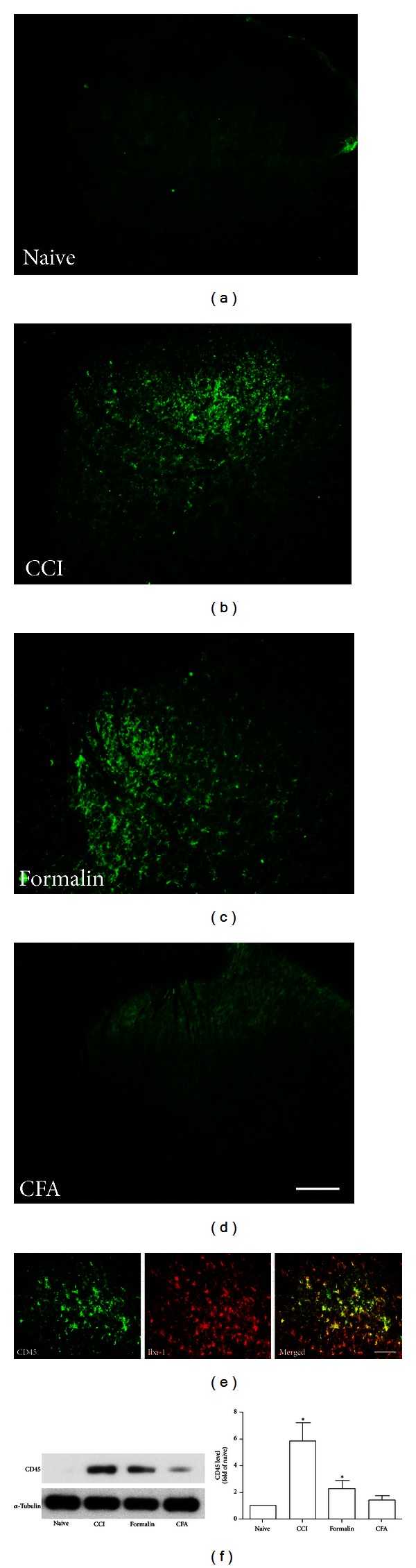
Significant increased expression of CD45 was found in the ipsilateral side of spinal cord of CCI and formalin-injected rats and weak expressed in CFA-injected rats ((a)–(d); Scale bar, 100 *μ*m). Double labeling with Iba-1 confirmed that the cell expressing CD45 is microglia ((e); Scale bar, 50 *μ*m). Representative bands and quantification of western blot analysis (f) showed significantly increased CD45 protein level in the lumbar spinal cord after CCI injury and formalin injection, not after CFA injection.

**Figure 3:**
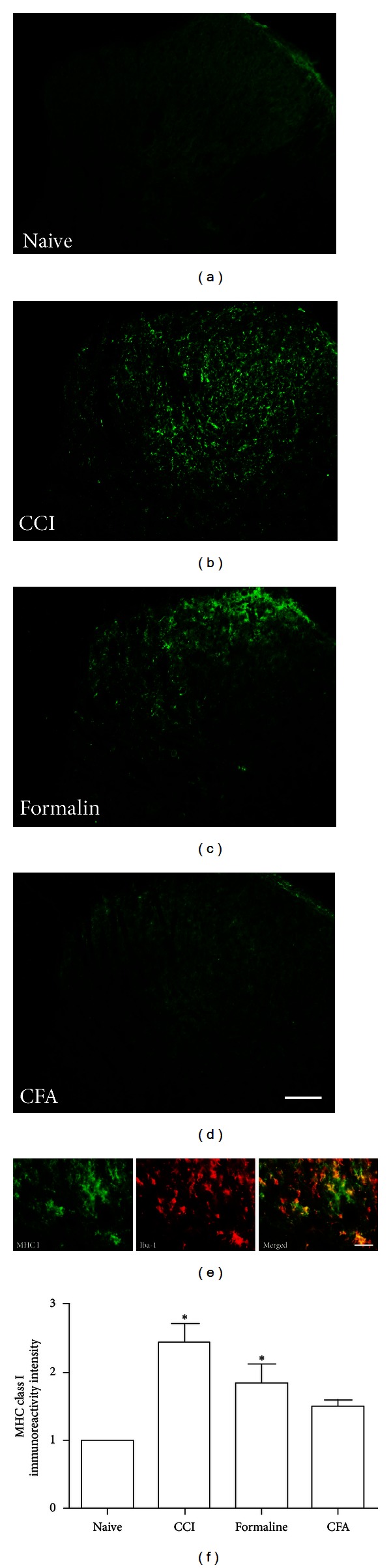
Significant upregulation of MHC class I expression was found in the spinal cord of CCI and formalin-injected rats but weak expressed in CFA-injected rats ((a)–(d) Scale bar, 100 *μ*m). MHC class I positive cells were colocalized with Iba-1 ((e) Scale bar, 30 *μ*m). The intensity of MHC class I immunoreactivity was quantitatively compared among four groups (f). *P < 0.05, as compared with the naïve group.

**Figure 4:**
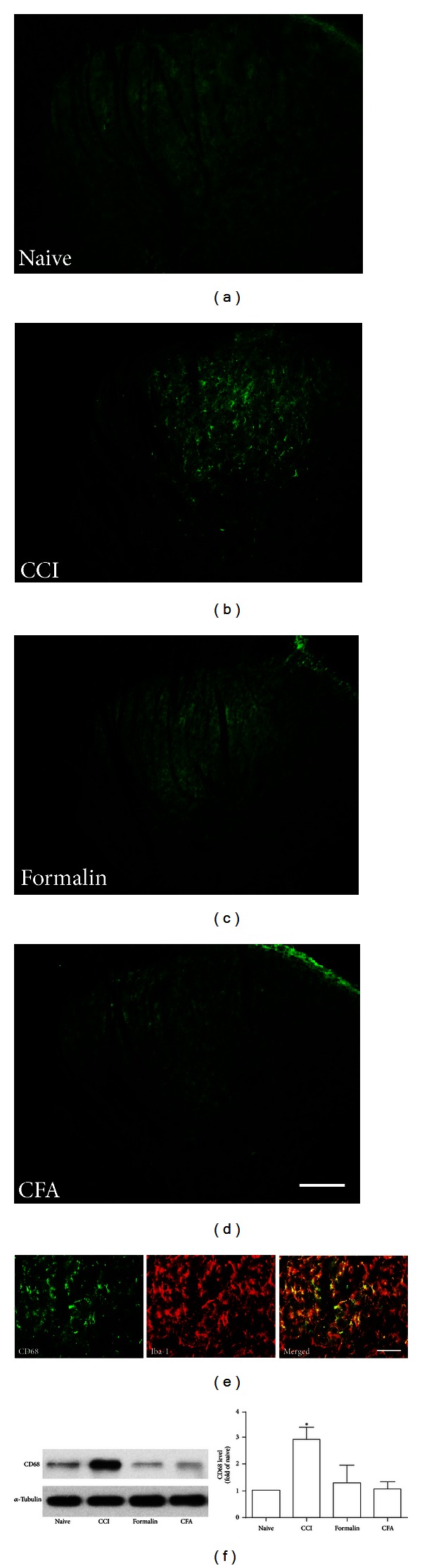
A significant increase of CD68 expression was present only following CCI injury, which was weak after formalin and CFA injection ((a)–(d); Scale bar, 100 *μ*m). CD68 positive cells were colocalized with Iba-1 ((e); Scale bar, 50 *μ*m). Representative bands of Western blot and quantification of Western blot analysis (f) showed increased level of CD68 in CCI group. *P < 0.05 as compared with the naïve group, *n* = 4.

Our previous study demonstrated that peripheral formalin injection induced phenotypic changes of microglia with distinct upregulation of CD45 and MHC class I antigens [28]. In the present study, CD45 and MHC class I immunoreactivity were also significantly upregulated on day 7 in the ipsilateral side following CCI injury and formalin injection (Figures 2 and 3). There was a very low basal expression of these two molecules in the CFA model (Figures 2 and 3). We also found that CCI injury induced significant expression of CD68 by immunofluorescence, but not seen in the formalin and CFA groups (Figure 4). The above observation was further supported by western blot analysis (Figures 2(f) and 4(f)). Because a well-proven anti-rat MHC class I antibody for western blot was not available, the immunofluorescence intensity was measured with quantification analysis instead of western blot (Figure 3(f)).

To identify the cell types, we performed double immunostaining with several cell-specific markers: NSE (neurons), GFAP (astrocytes), and Iba-1 (microglia). CD45, CD68, and MHC class I did not colocalize with either NSE or GFAP but mostly colocalized with Iba-1 (Figures 2(e), 3(e), and 4(e)). 

## 4. Discussion

In our previous study, we investigated the possible induction of several microglial surface immune molecules in the spinal cord and found that CD45 and MHC class I antigens were significantly upregulated following peripheral formalin injection into the rat hind paw [28]. The time course of the increased level of CD45 and MHC class I immunoreactivity demonstrated that both CD45 and MHC class I upregulations were evident on day 3 with the peak expression on day 7. In the present study, we compared CD45, CD68 and MHC class I expression on day 7 in three animal pain models using immunohistochemistry and quantitative western blot analysis. Our results clearly indicated that different peripheral tissue injury produced differential phenotypic changes associated with spinal microglial activation. Peripheral nerve injury, not inflammation, induced the activated microglia to acquire these immunomolecular phenotypic changes. 

Spinal microglial activation has been extensively reported in almost all animal pain models using OX-42 immunohistochemical marker and mitogen-activated protein kinases (MAPKs) such as phosphorylated p38 (p-p38) [4–6]. Significant microglial activation with morphological changes was evident as early as 2-3 days, maximal at 7 days, after peripheral tissue injury [7–13, 33]. However, inflammatory pain models produced by intraplantar injection of CFA [31, 34] and muscle pain by intramuscular acidic saline injection [35] caused only minor or no microglial activation. Microglia could be rapidly activated without morphological changes following peripheral inflammation as indicated by the increased expression of p38 MAP kinase in the spinal microglia [36]. It was reported that microglia might undergo at least two distinct stages of activation on the basis of their morphological and phenotypic changes [28]. (1) Early-activated microglia display a “resting” ramified morphology with a relatively small cell body and weakly express molecules normally present in other haematopoietic lineages, such as CD45, MHC class I antigen, and other immunomolecules. (2) Late-activated microglia show upregulation of CD45 and MHC class I and a morphology characterized by the hypertrophic cell body and the shortening of cellular processes. In the present study in CCI model, activated microglia showed the strongest upregulation of immune molecules, such as CD45, CD68, and MHC class I with a robust degree of morphological change being observed, but CFA-induced inflammation did not. Peripheral formalin injection also produced significant microglial activation and upregulation of some immune molecules. As we discussed in our previous publication [37], the formalin test is both a short-term inflammatory and a long-term nerve injury pain model. A 5% formalin injection damages the local nociceptive receptors at the injection site and results in the loss of responses to thermal and mechanical stimuli [31]. Peripheral formalin injection damaged nerve endings and produced spinal microglial activation. However, the damage was mild if compared to CCI injury, the spinal microglial activation was not stronger than CCI and did not induce significant upregulation of CD68. These findings suggested that spinal microglial activation and cell surface immune molecular expression were dependent on the pathogenic stimulus administered; peripheral nerve injury caused the late-activated microglia to acquire immune phenotypic and morphological changes. However, molecular mechanisms for the spinal microglial activation by peripheral nerve injury are still unknown. Inflammation/immune response at the site of the injured sciatic nerve has been well elucidated, but there is no report on the inflammatory status of the ipsilateral side of spinal nerve following peripheral injection of CFA or formalin into the animal's hind paw. Whether inflammation/immune response of the ipsilateral side of spinal nerve is an essential trigger for the spinal microglial activation is worth further doing. 

## 5. Conclusions

With different pain conditions, microglia could exist in differential statuses of activation. The immunomolecular and phenotypic changes of microglia are associated with nerve injury in most cases. For this reason, elucidation of the different activation modalities of microglia could push us to find more specific and promising targets for inhibiting microglial activation associated pathological pain.
